# The Swiss Haemophilia Registry–Report From the First 8 Years

**DOI:** 10.1111/hae.70294

**Published:** 2026-04-21

**Authors:** Alessandra Bosch, Lorenzo Alberio, Pierre Fontana, Lukas Graf, Johanna A. Kremer Hovinga, Nicolas von der Weid, Mattia Rizzi, Manuela Albisetti, the Swiss Haemophilia Network

**Affiliations:** ^1^ Department of Haematology University Children's Hospital Zurich Zurich Switzerland; ^2^ Service and Central Laboratory of Haematology Lausanne University Hospital (CHUV) and University of Lausanne (UNIL) Lausanne Switzerland; ^3^ Division of Angiology and Haemostasis, Faculty of Medicine University Hospitals of Geneva and Geneva Platelet Group Geneva Switzerland; ^4^ Eastern Switzerland Haemophilia and Haemostasis Centre Centre For Laboratory Medicine St. Gallen Switzerland; ^5^ Department of Hematology and Central Hematology Laboratory Inselspital, Bern University Hospital, University of Bern Bern Switzerland; ^6^ Department of Pediatric Hematology‐Oncology University Children's Hospital Basel (UKBB) and University of Basel Basel Switzerland; ^7^ Paediatric Haematology‐Oncology Unit Lausanne University Hospital (CHUV) and University of Lausanne (UNIL) Lausanne Switzerland; ^8^ Pediatric Institute of Southern Switzerland Ente Ospedaliero Cantonale Bellinzona Switzerland

**Keywords:** hemophilia A, hemophilia B, rare diseases, registries, von Willebrand diseases

## Abstract

**Introduction:**

Patient registries capture disease related information and provide a valuable source for real‐world data on rare diseases and their management. The Swiss Haemophilia Registry (SHR) was established in 2015 on the basis of a new Swiss federal human research act. It includes patients with inherited bleeding disorders, namely haemophilia A and B, von Willebrand disease (VWD), other rare bleeding disorders, and platelet function disorders.

**Aim:**

To describe the bleeding disorder landscape in Switzerland.

**Methods:**

The SHR is an observational, prospective, longitudinal, multi‐centre national registry. Individual patient data is collected annually and includes patient demographics, comorbidities, bleeding events and treatment.

**Results:**

By 2023, 929 patients were included in the SHR, with 60% diagnosed with haemophilia A, 17% with haemophilia B, and 15% with VWD. The cohort was predominantly male (87%), and 75% were adults. Median follow‐up was 5.8 years (IQR 3.35–7.22). The prevalence of target joints in 2023 was 2%, with no affected children. Annual inhibitor prevalence in haemophilia patients was 1–2%. The SHR illustrates clearly the transition of prophylaxis products from plasma‐derived to extended half‐life factor products, and non‐factor products, mirroring the global treatment evolution, and trends in individualised and patient‐centred haemophilia management.

**Conclusion:**

The SHR provides real‐world evidence on haemophilia care in Switzerland and documents major improvements in treatment and patient outcomes over the past decade. Future expansion will be more inclusive of VWD, rare bleeding disorders, and specifically women with bleeding disorders. This will enhance the value of the SHR as a comprehensive national resource.

## 1 Introduction

Patient registries are essential for collecting real‐world, longitudinal data on rare diseases, including prevalence, incidence, treatment patterns, and outcomes. These areas are often not addressed by clinical trials. International bleeding disorder registries enable comparison of care models, health systems, and clinical practices, helping to identify factors that influence outcomes in rare bleeding disorders. These registries support research, improve understanding of disease variability, and inform best practices in patient management [1, 2, 3].

**Plain Language Summary**

Patient registries collect real‐world information about people with rare diseases. The Swiss Haemophilia Registry (SHR), created in 2015, collects yearly health information from people in Switzerland with bleeding disorders. By 2023 it included 929 patients, most with haemophilia. The registry shows major improvements in care, with a shift to newer, more effective treatments. Only a small number of patients had complications such as serious joint problems (2%) or treatment‐related inhibitors (1–2%). Overall, the SHR demonstrates that haemophilia care in Switzerland has improved significantly in the past decade. Future efforts will focus on including more people with bleeding disorders, especially von Willebrand disease, other rare bleeding disorders, and women with bleeding disorders, to make the registry an even more valuable national resource.

Hereditary bleeding disorders are rare diseases and good quality data is needed to improve clinical care. Over the past decades there has been a tremendous development and improvement of therapy‐options especially for haemophilia A and B. These have included the transition from plasma‐derived products to recombinant products, from standard to extended half‐lives, and more recent developments including non‐factor products, rebalancing agents, and gene therapy [4, 5].

The old Haemophilia Registry in Switzerland created in 2002 had to be shut down due to a new federal act on research involving human beings (HRA) that came into effect in Switzerland in 2014 [6]. The old registry no longer complied with the new regulations, and reapproval by the different regulatory authorities and re‐consenting of participants was required. Therefore, the Swiss Haemophilia Registry (SHR) was established in 2015 under the Swiss Haemophilia Network, an interprofessional association. The SHR is designed to capture comprehensive data on clinical manifestations, therapeutic interventions, and comorbid conditions—including inhibitor development, viral infections, and joint disease—across the spectrum of rare bleeding disorders. By adhering to internationally accepted quality criteria for registry governance, data integrity, and interoperability, the registry aims to support research, inform clinical practice, and guide health policy to improve outcomes for individuals affected by these rare conditions in Switzerland.

This first report from the SHR aims to add to the real‐world data on rare bleeding disorders, increasing the evidence on bleeding disorders’ care and how it has evolved over the past decade.

## 2 Methods

### 2.1 Study Design and Setting

The SHR is an observational, prospective, longitudinal, multi‐centre national registry of patients with haemophilia and other inherited bleeding disorders.

Follow‐up data is collected annually through a web‐based Research Electronic Data Capture tool (REDCap), which is installed on the server of the Clinical Trial Unit (CTU) of the University of Bern, Switzerland (Department of Clinical Research) [7, 8]. An independent annual data monitoring has been set up for quality control and assurance of data completeness. This monitoring is performed by an experienced independent haemophilia nurse who visits each haemophilia treatment centre in person annually, conducting random sampling checks to verify data accuracy and completeness, and addressing any questions or uncertainties from local staff on‐site. This report summarizes major findings from the data analysis covering the period 2015 to 2023, and is reported according to the Strengthening the Reporting of Observational Studies in Epidemiology statement (STROBE) [9]. Ethics approval from each canton's ethics review board was obtained for the registry.

### 2.2 Participants

All haemophilia treatment centres in Switzerland recruit and include patients with bleeding disorders into the SHR after informed consent is obtained from each patient. The eligibility criteria for each bleeding disorder are included in Table 1. The age of the population is calculated as per the year 2023 (data cut‐off). The follow‐up time is calculated by date of registry entry (consent) to last documented annual follow‐up.

**TABLE 1:** Demographics of all included patients in the Swiss Haemophilia Registry from 2015–2023. Some patients were identified to have more than one bleeding disorder. Abbreviations: F, factor; VWF, von Willebrand factor.

Demographics	*N* = 929
Sex	
Female	119/929 (13%)
Male	810/929 (87%)
**Age**	37 (20, 56)
<18 years	230/929 (25%)
≥18 years	699/929 (75%)
**Bleeding disorder**	
Haemophilia A	556/929 (60%)
Mild (FVIII:C > 5%)	248/556 (45%)
Moderate (FVIII:C 1–5%)	79/556 (14%)
Severe (FVIII:C < 1%)	229/556 (41%)
Haemophilia B	162/929 (17%)
Mild (FIX:C > 5%)	76/162 (47%)
Moderate (FIX:C 1–5%)	41/162 (25%)
Severe (FIX:C < 1%)	45/162 (28%)
Von Willebrand disease	136/929 (15%)
Type 1 (VWF‐antigen or VWF‐activity < 30%)	38/136 (28%)
Type 2	89/136 (65%)
2A	55/89 (62%)
2B	11/89 (12%)
2M	15/89 (17%)
2N	4/89 (4.5%)
Unknown	4/89 (4.5%)
Type 3	9/136 (6.6%)
Fibrinogen disorder	20/929 (2.2%)
Hypofibrinogenemia (Fibrinogen < 0.1 g/L)	8/20 (40%)
Afibrinogenemia (qualitative disorder)	9/20 (45%)
Dysfibrinogenemia (Fibrinogen <1.5 g/L)	3/20 (15%)
**Other Rare Bleeding Disorders**	
Factor V deficiency (FV:C < 10%)	6/929 (0.6%)
Factor VII deficiency (FVII:C < 10%)	19/929 (2.0%)
Factor X deficiency (FX:C < 10%)	4/929 (0.4%)
Factor XI deficiency (FXI:C < 10%)	23/929 (2.5%)
Factor XIII deficiency (FXIII:C < 10%)	16/929 (1.7%)
Other factor deficiency	3/929 (0.3%)
**Platelet disorder**	
Glanzmann thrombasthenia	7/929 (0.8%)
Bernard Soulier disease	1/929 (0.1%)

*Note*: *n* / *N* (%); Median (IQR).

All registered patients were included in the analysis regardless of attendance at annual follow‐up visits. Patients without complete annual data in a given year contribute data only for years in which follow‐up was documented.

### 2.3 Variables

Data collected for each patient is de‐identified. Only registered staff from each haemophilia treatment centre have password‐access to the REDCap case reporting forms and can only access their own centre's patient data. At registry inclusion, year of birth, sex assigned at birth, baseline bleeding disorder diagnosis, severity of haemophilia (mild, moderate, severe) are entered. Clinical data is entered by trained centre staff during annual in‐person follow‐up visits at the haemophilia treatment centre. Patients do not directly interact with the registry database; no patient‐facing online tools or mobile applications are used for data entry into the registry.

Annual follow‐up data include bleeding disorder related treatments (primary/secondary/tertiary prophylaxis, on‐demand treatment), and the type of treatment (plasma‐derived standard half‐life [pSHL] concentrates, recombinant SHL [rSHL] products, recombinant extended half‐life [rEHL] products, prothrombin complex concentrates [PCC], bypassing agents [rFVIIa, FEIBA], Non‐factor therapies [e.g. emicizumab], liver transplant, or study medication for treatment within clinical trials). Number of bleeding events and surgeries requiring factor replacement are also documented annually.

Target joints are recorded and defined as spontaneous bleeding into a joint three or more times in a 6‐month period. Multiple target joints are defined as two or more target joints.

Inhibitor outcomes are defined as negative (< 0.6 Bethesda Units (BU/ml)), low titre (0.6 to 5.0 BU/ml), high titre (> 5.0 BU/ml), and are recorded as inhibitor status at end of year.

For comorbidities the diagnosis of hepatitis B/C/HIV (human immunodeficiency virus), currently active disease (PCR positivity), their treatment and whether the disease was transmitted from contaminated blood products is recorded at registry entry and if the status changes over time.

Where applicable, antithrombotic treatment was recorded per year with indication of anticoagulation and/or anti‐platelet drug, and the agent used (direct oral anticoagulant [DOAC], vitamin K antagonist, low molecular weight heparin [LMWH], unfractionated heparin, acetylsalicylic acid [ASA], anti‐P2Y_12_, other).

### 2.4 Statistical Analysis

This report analyses data on patients entered into the registry, from its establishment in January 2015 to December 2023. The year 2023 is chosen as cut‐off year due to data completeness and complete monitoring. Descriptive statistics are used to report demographic data on the Swiss Haemophilia Treatment Centres, the patients, and types of bleeding disorders with frequencies, medians, and 25^th^ to 75^th^ percentiles (interquartile range, IQR).

## 3 Results

**FIGURE 1:**
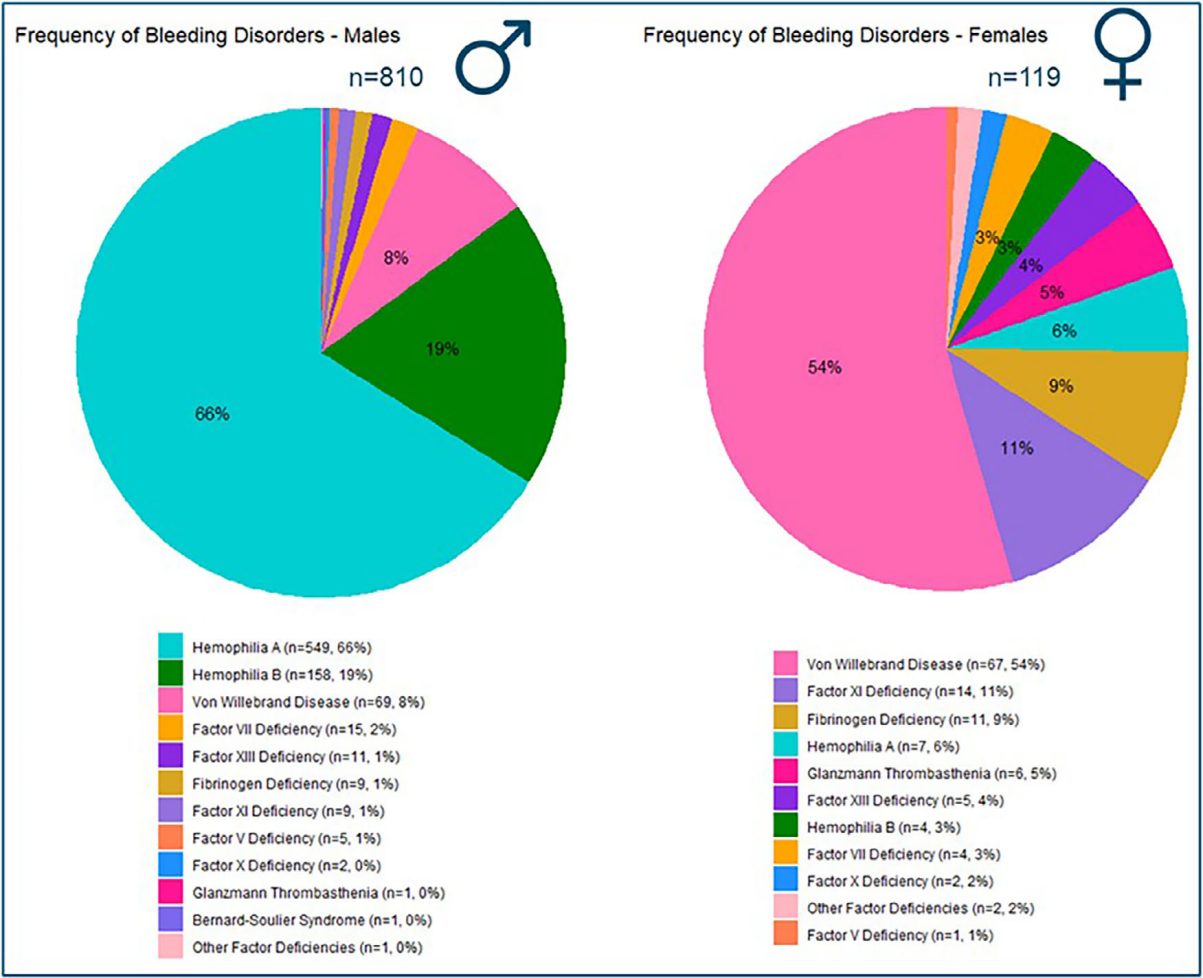
Frequency of bleeding disorders by sex.

As of December 2023, a total of 929 adult and paediatric patients were included in the SHR from eleven Swiss haemophilia treatment centres. In the year 2023, complete annual data was collected on 860 (93%) out of the 929 patients. Of all included patients, 556 (60%) had haemophilia A, 162 (17%) had haemophilia B, 136 (15%) had von Willebrand disease (VWD), and 75 (8%) had other rare bleeding disorders. Based on the 2022 annual national reporting survey from all haemophilia treatment centres in Switzerland, this represents 85%–90% of all haemophilia patients in Switzerland. The frequency, severity and / or subtype of all included bleeding disorders is shown in Table 1. Of patients with haemophilia A, 45% had mild, 14% moderate, and 41% severe disease. Of patients with haemophilia B, 47% had mild, 25% moderate, and 28% severe disease. In addition, the frequency of haemophilia and other bleeding disorders in the registry by sex are shown in Figure 1.

In the SHR cohort 87% were male and 75% were older than 18 years (Table 1). The median follow‐up time was 5.8 years (IQR 3.35–7.22) per patient with a total follow‐up time of 4850 patient years within the registry. The sex distribution of bleeding disorders differed markedly: among males (*n* = 810), haemophilia A was the predominant diagnosis (66%), followed by haemophilia B (19%) and VWD (8%). Among females (*n* = 119), VWD was the most frequent diagnosis (54%), followed by Factor XI deficiency (11%) and fibrinogen deficiency (9%), with haemophilia A and B accounting for 6% and 3% respectively.

### 3.1 Haemophilia Treatment

**FIGURE 2:**
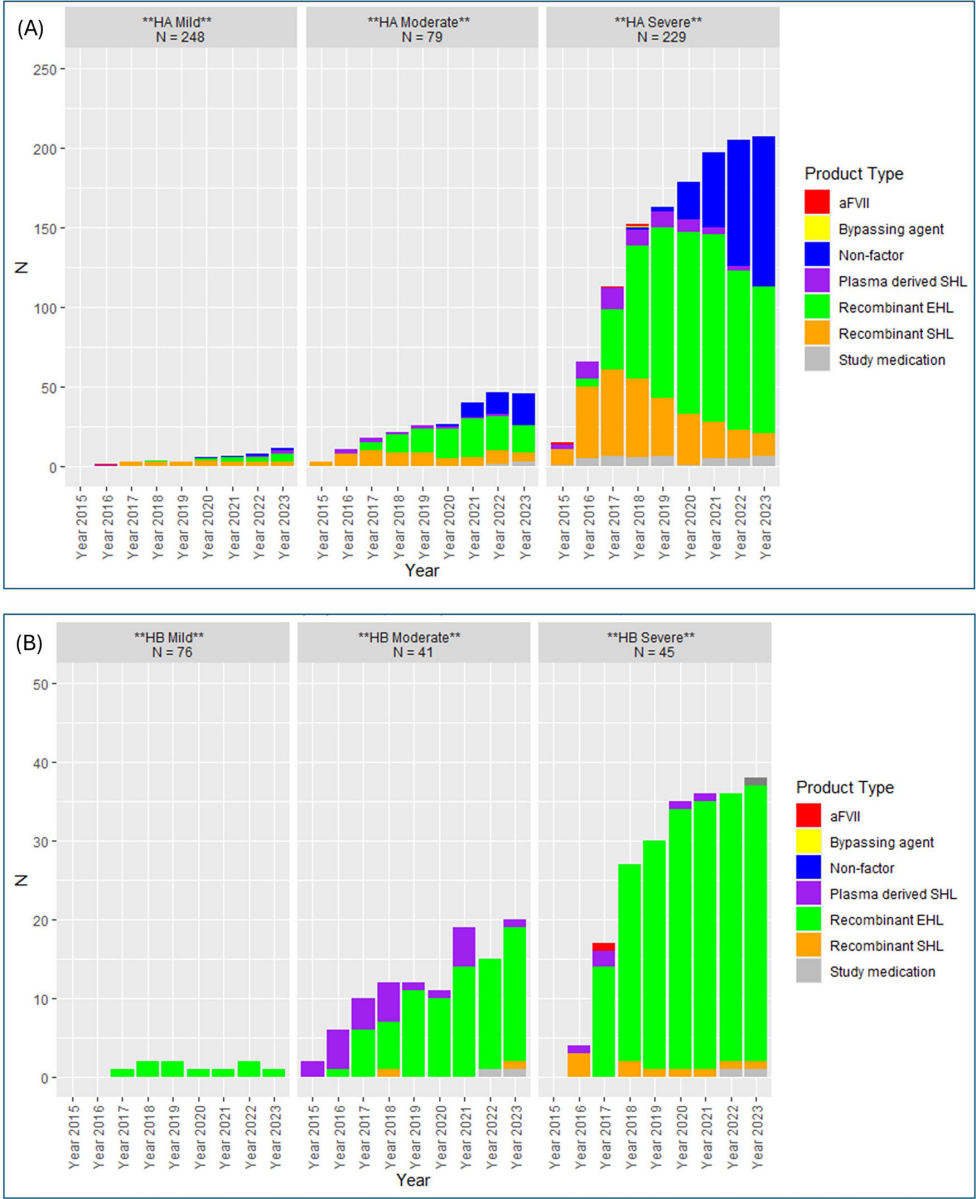
Patients receiving prophylaxis over time by severity of disease mild/moderate/severe for (A) Haemophilia A, (B) Haemophilia B. Type of prophylaxis products are shown for total number of patients respectively. Abbreviations: HA, Haemophilia A; HB, Haemophilia B.

The number of patients with haemophilia A and B receiving prophylaxis treatment and type of product is shown from 2015 to 2023 for mild, moderate and severe disease respectively (Figure 2). Over the observation period, a clear shift from plasma‐derived and SHL‐recombinant products towards recombinant EHL products and non‐factor therapies was observed, most prominently in severe haemophilia (Figure 2). No liver transplants or gene therapies were performed in 2015–2023.

For the year 2023, 98% (205 of 210) of patients with severe haemophilia A and 93% (38 of 41) of patients with severe haemophilia B received prophylaxis, while 16% (33 of 207) of patients with other bleeding disorders received prophylaxis. The use of prophylaxis and of on‐demand therapy across age cohorts and for severe/moderate/mild haemophilia is shown in the Figure S1. Prophylaxis rates were consistently high across all paediatric and adult age groups in severe haemophilia A and B, whereas on‐demand therapy was more prevalent in older adult patients, and in patients with mild and moderate haemophilia A and B (Figure S1).

### 3.2 Bleeding Events

**FIGURE 3:**
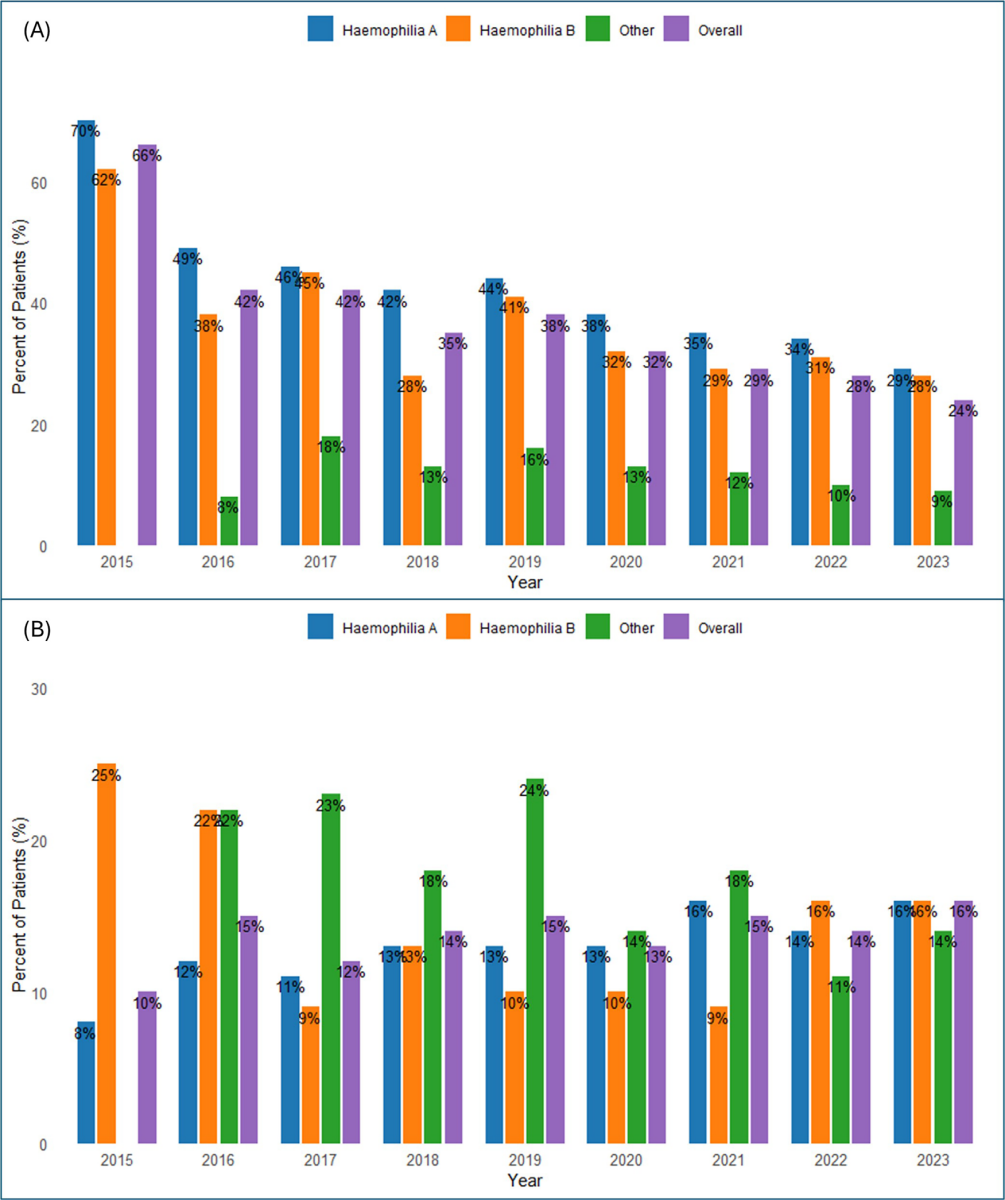
Annual treated bleeds and surgeries by year and by bleeding disorder. (A) Patients with treated bleeds(B) Patients with surgeries requiring product.

In 2023, 24% (208 of 860) of patients received additional factor products for a total of 460 bleeding events, and 16% (135 of 860) of patients required factor products for surgeries. The percentage of patients with treated bleeds decreased over time across all bleeding disorder groups. In 2015, 70% of patients with haemophilia A, 62% with haemophilia B, and 65% overall experienced treated bleeds, decreasing to 29%, 28%, and 24% respectively by 2023 (Figure 3A). Among non‐haemophilia diagnoses, Glanzmann thrombasthenia consistently showed the highest percentage of patients with treated bleeds, while fibrinogen deficiency and severe factor VII deficiency also showed notable rates across multiple years. Other rare bleeding disorders showed lower and more variable rates, likely reflecting smaller patient numbers within these groups (Figure S5).

The percentage of patients requiring factor products for surgeries remained relatively stable over time, ranging between approximately 10–25% across all bleeding disorder groups (Figure 3B). Neither the type of bleeding event nor the type of surgery is reported in the SHR.

Of patients with haemophilia A and B in 2023, 13 had at least one active target joint (prevalence of 2%), whereas 5 had multiple active target joints (prevalence of 0.8%). The number of patients with target joints peaked in 2018 (10.6% of patients with at least one target joint among adults). No paediatric patient was affected with a target joint in 2023. The trend of target joints over time is shown in Figure S2.

### 3.3 Complications of Treatment

The number and prevalence of haemophilia patients with inhibitors per year is shown in Table 2. Low‐titre inhibitors were consistently more frequent than high‐titre inhibitors throughout the observation period. The total number of patients with inhibitors ranged from 1 (2015) to 9 (2018), with an overall inhibitor prevalence ranging from 1.11% (2022) to 2.08% (2015) (Table 2).

**TABLE 2:** Inhibitor prevalence and number of patients with active low and high titre inhibitors per year.

Low and high titre inhibitors per year—counts and prevalence				
Year	Low‐titre (*n*)	High‐titre (*n*)	Total with inhibitor (*n*)	Inhibitor prevalence per year (%)
2015	1	0	1	2.08
2016	4	0	4	1.59
2017	6	1	7	1.95
2018	8	1	9	1.96
2019	7	2	9	1.80
2020	7	3	10	1.84
2021	6	3	9	1.50
2022	5	2	7	1.11
2023	7	2	9	1.38

**TABLE 3:** Comorbidities in children and adults with bleeding disorders.

Comorbidities	<18 years *N* = 229	>18 years *N* = 691
Hepatitis B		
Diagnosis of hepatitis B	0/229 (0%)	83/691 (12%)
Hepatitis B due to contaminated blood products	0/229 (0%)	38/691 (6%)
Active hepatitis B status	0/229 (0%)	10/691 (1%)
**Hepatitis C**		
Diagnosis of hepatitis C	0/229 (0%)	167/691 (24%)
Hepatitis C due to contaminated blood products	0/229 (0%)	117/691 (17%)
Active hepatitis C status	0/229 (0%)	14/691 (2%)
**HIV**		
Diagnosis of HIV	0/229 (0%)	19/691 (3%)
HIV due to contaminated blood products	0/229 (0%)	15/691 (2%)

No paediatric patients were affected by hepatitis B, C or HIV. Among adult patients, 12% had a diagnosis of hepatitis B, of whom 5.6% acquired it through contaminated blood products, and 1.4% had active hepatitis B. Hepatitis C was diagnosed in 24% of adult patients, with 17% having acquired it through contaminated blood products, and 2% having current active hepatitis C. HIV was diagnosed in 3% of adult patients, with 2% attributable to contaminated blood products (Table 3).

### 3.4 Other Health‐Related Information Captured

The distribution of patients among study centres within Switzerland is shown in Figure S3.

The number of patients receiving antithrombotic treatment increased steadily from 3 patients in 2016 to 37 patients in 2023. The most common indication was perioperative thromboprophylaxis, and the most frequently used agent was LMWH. The number of patients, indication and type of antithrombotic treatment over time are shown in Figure S4.

## 4 Discussion

This first evaluation of the SHR provides a comprehensive overview of the national bleeding disorder landscape over the eight years since its establishment in 2015. The registry primarily includes patients with haemophilia A and B, representing 86% of the Swiss Haemophilia population. The distribution of disease severity within the SHR, is comparable to the distribution within the World Federation of Hemophilia (WFH) global survey of high‐income countries. This underscores the representativeness of the SHR haemophilia population [10].

Analysed data from the registry reflects the therapeutic evolution, showing a transition from pSHL concentrates to rEHL products and non‐factor therapies over time. This shift has been accompanied by a clear reduction in target joints, highlighting the benefits of prophylaxis, which has become standard of care not only for patients with severe but also increasingly for those with moderate haemophilia [4, 11, 12]. The implementation of prophylaxis for severe and moderate haemophilia across all age groups is evident compared to reports from the old haemophilia registry in Switzerland [6].

Age‐dependent outcome differences are evident. Older patients in the SHR present with more comorbidities and joint damage, reflecting the limited treatment options available in earlier decades and possibly hesitance to adopt new treatment options, whereas younger patients benefit from early prophylaxis and access to novel therapies. The near absence of transfusion‐related infections such as hepatitis and HIV in children demonstrates the success of improved product safety, antiviral treatments and vaccines. These observations align with international findings and underline the progress achieved in haemophilia care [13].

Despite the strong representation of haemophilia, other rare bleeding disorders and female haemophilia carriers remain underrepresented in the SHR. Compared to international prevalence estimates and the World Federation of Hemophilia Annual Global Survey, the number of patients with rare bleeding disorders and symptomatic female carriers in the SHR is substantially lower than expected [10, 14]. For example, U.S. registry data show that cases of certain rare bleeding disorders are underreported by 36–94% relative to Orphanet prevalence estimates, and only a minority of female carriers with clinically significant bleeding are captured in national registries [15, 16, 17]. This limits a comprehensive understanding of the local bleeding disorder population and underlines the need for registry expansion. More inclusive practices of patients with von VWD and rare bleeding disorders, as well as haemophilia carriers, women with haemophilia, and other bleeding disorders would strengthen the registry's representativeness and support efforts to address diagnostic and therapeutic gaps [5, 14, 18, 19].

A current focus of the SHR is the systematic documentation of haemostatic agents in relation to prophylaxis, bleeding events and surgical interventions. These data are of particular importance given the rapid expansion of treatment options in recent years, now also with several novel agents which have entered the market most recently. Monitoring real‐world factor consumption will be essential for evaluating treatment patterns, adherence to guidelines and the economic impact of therapy choices [3].

Beyond clinical insights, registry data have important implications for health policy. In rare diseases such as haemophilia, real‐world data provide critical evidence for discussions with healthcare payers. Demonstrating that these expensive therapies can reduce bleeding frequency, improve joint health, reduce the number of joint interventions, and increase the work ability of individuals supports their long‐term sustainability within national healthcare systems [1, 3].

Looking ahead, the advent of ultra‐long‐acting factor products, rebalancing agents, and gene therapy will further transform haemophilia management. Continuous data collection within the SHR will be essential to evaluate the long‐term effectiveness, safety and cost‐effectiveness of these emerging therapies and ensure optimal patient care in a rapidly evolving therapeutic landscape.

The SHR data can be compared with data from other high‐income countries, although this requires careful interpretation. Comparability depends on shared structural features, including comprehensive access to care, specialized treatment centres, and widespread availability of advanced therapies. Differences in registry design, data completeness, outcome definitions (e.g., annualized bleeding rate, inhibitor status), patient selection, and reimbursement systems may also introduce bias. Reimbursement policies may substantially influence therapeutic switching: it may determine whether switching occurs and to which therapies patients switch. In Switzerland, physicians retain substantial autonomy to select the most appropriate therapy among reimbursed options, without strict central recommendations or obligations. This is in contrast to countries with a tender system or with restricted availability of therapeutic options. This physician autonomy, within a framework of comprehensive reimbursement, supports more individualized treatment decisions while maintaining access to the full spectrum of available therapies [20]. Efforts within the Society of Thrombosis and Haemostasis Research e.V.’s (GTH) member countries particularly Germany, Austria, and Switzerland, aim to improve harmonization and comparability. These include the establishment of standardized certification processes for haemophilia centres, progressive development of structured national registries, and expert‐driven coordinated approaches for the management of emerging therapies such as gene therapy [21, 22, 23, 24]. Nevertheless, full cross‐country harmonization of data acquisition remains an evolving objective.

Treatment practices and outcomes of the SHR are consistent with those of other high‐income countries. Evidence from the PedNet cohort, together with national datasets from Germany and Austria, indicates a high level of implementation of contemporary standards of care, particularly with respect to early initiation of prophylaxis and structured longitudinal follow‐up. These findings suggest alignment with treatment patterns reported in other high‐income settings, as reflected in global data from the World Federation of Hemophilia registry, although direct comparative analyses remain limited [2, 11, 12, 13, 22]. In line with this, European registry data from the European Haemophilia Safety Surveillance also demonstrate a substantial shift toward the use of innovative therapies, including emicizumab for haemophilia A and rEHL products for haemophilia B, consistent with evolving standards of care across European settings [25].

Several limitations should be acknowledged. Although all national haemophilia treatment centres contribute to the SHR, patient coverage currently reaches 85–90% of the expected population with haemophilia, which may introduce selection bias. Treatment bias is also likely, as older patients represent cohorts exposed to less effective treatment, sometimes for very long periods of time. Furthermore, the registry relies on accurate and complete data entry, and underreporting or exclusion criteria may affect certain subgroups, particularly those with rare bleeding disorders or milder phenotypic disease (e.g. low von Willebrand factor, mild platelet disorders, and mild factor deficiencies). Reasons for therapy changes and the clinical rationale for treatment decisions are not recorded in the SHR, limiting conclusions about treatment decision‐making patterns. Nonetheless, the overall alignment of SHR findings with data from comparable international registries supports the validity and generalizability of the results.

In conclusion, the SHR provides valuable real‐world evidence on haemophilia care in Switzerland and documents major improvements in patient outcomes over the past decade. The registry serves as a robust platform for monitoring treatment trends, benchmarking national practices, and guiding healthcare planning. Future expansion to include all bleeding disorders, including VWD, rare bleeding disorders, and specifically women with bleeding disorders, will enhance its value as a comprehensive national resource.

## Funding

Registry received financial support from Bayer, Biotest, CSL Behring, NovoNordisk, Octapharma, Pfizer, Roche, Sobi, Takeda. AB is supported by research grants from the “Claus Cramer Foundation”, “Filling the gap program” and “Walter and Gertraud Siegenthaler Foundation” from the University of Zurich, Switzerland.

## Ethics

Ethics approval from responsible cantonal ethics review committees was obtained for the registry.

## Consent statement

Signed informed consent was obtained from each patient before inclusion into the registry.

## Conflicts of Interest

Alessandra Bosch: received travel grants from Novonordisk, Roche, Sobi; Institutional reimbursement fees from CSL Behring, Novonordisk, Roche, Sobi, Takeda. Lorenzo Alberio: received travel grants from Sobi and NovoNordisk, as well as fees from NovoNordisk, Pfizer, Roche, Sanofi, and Sobi. Pierre Fontana: received travel grants from Sobi, NovoNordisk and Takeda and fees from Sobi and Roche. Lukas Graf: has received speaker's fees from Novo Nordisk, Roche, and Sobi; honoraria from Bayer, CSL Behring, Novo Nordisk, Pfizer, Roche, and Sobi; and research grant funding from Sobi and Roche. Johanna A. Kremer Hovinga: received travel support from CSL‐Behring, NovoNordisk, Roche, Sobi and Takeda. Institutional reimbursement for participation in advisory boards of from CSL‐Behring, NovoNordisk, Roche, Sobi and Takeda. The Project Interprofessional haemophilia care at EHCCC Interspatial Bern has received funding from CSL‐Behring, NovoNordisk, Roche and Sobi. Nicolas von der Weid: No conflicts of interest to declare. Mattia Rizzi: institutional reimbursement for consultancy services, speaker fees and/or travel grants from CLS Behring, NovoNordisk, Octapharma, Roche, Sobi and Takeda. Manuela Albisetti: received travel grants from NovoNordisk, Roche, Sobi; Institutional reimbursement fees from Novonordisk, Roche, Sobi.

## Supporting information




**Supporting Information Figure S1**: Percentage of SHR patients on prophalyxis (left panel) or treated on demand (right panel) in 2023. **Supporting Information Figure S2**: Number of patients with haemophilia A and B with one or multiple target joints in children and adults. **Supporting Information Figure S3**: Distribution of study centres for adults and children within Switzerland. **Supporting Information Figure S4**: Patients with hereditary bleeding disorders receiving anticoagulation or anti‐platelet therapy. Indication (panel A) and product used (panel B). **Supporting Information Figure S5**: Percent of patients with treated bleeds by year and by bleeding disorder.

## Data Availability

The data that support the findings of this study are available from the corresponding author upon reasonable request.
