# Supplementary material for: The Swiss Haemophilia Registry–Report From the First 8 Years
Source: Haemophilia. 2026 Apr 21;32(4):923–32. doi: 10.1111/hae.70294 (PMC13378617; doi:10.1111/hae.70294)

Appendix:

Supplementary Figure 1: Percentage of SHR patients on prophalyxis (left panel) or treated on demand (right panel) in 2023.

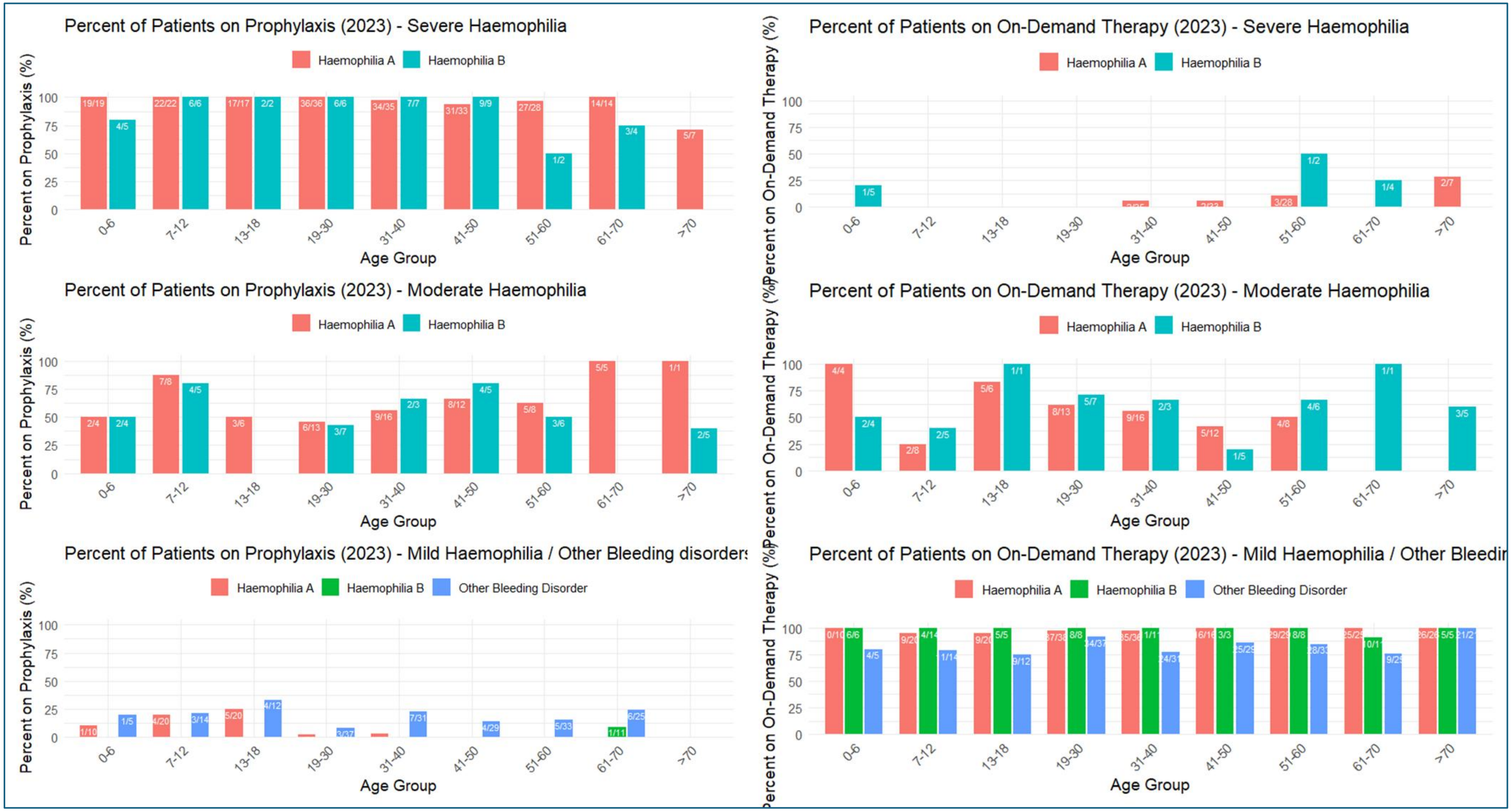

Supplementary Figure 2: Number of patients with haemophilia A and B with one or multiple target joints in children and adults.

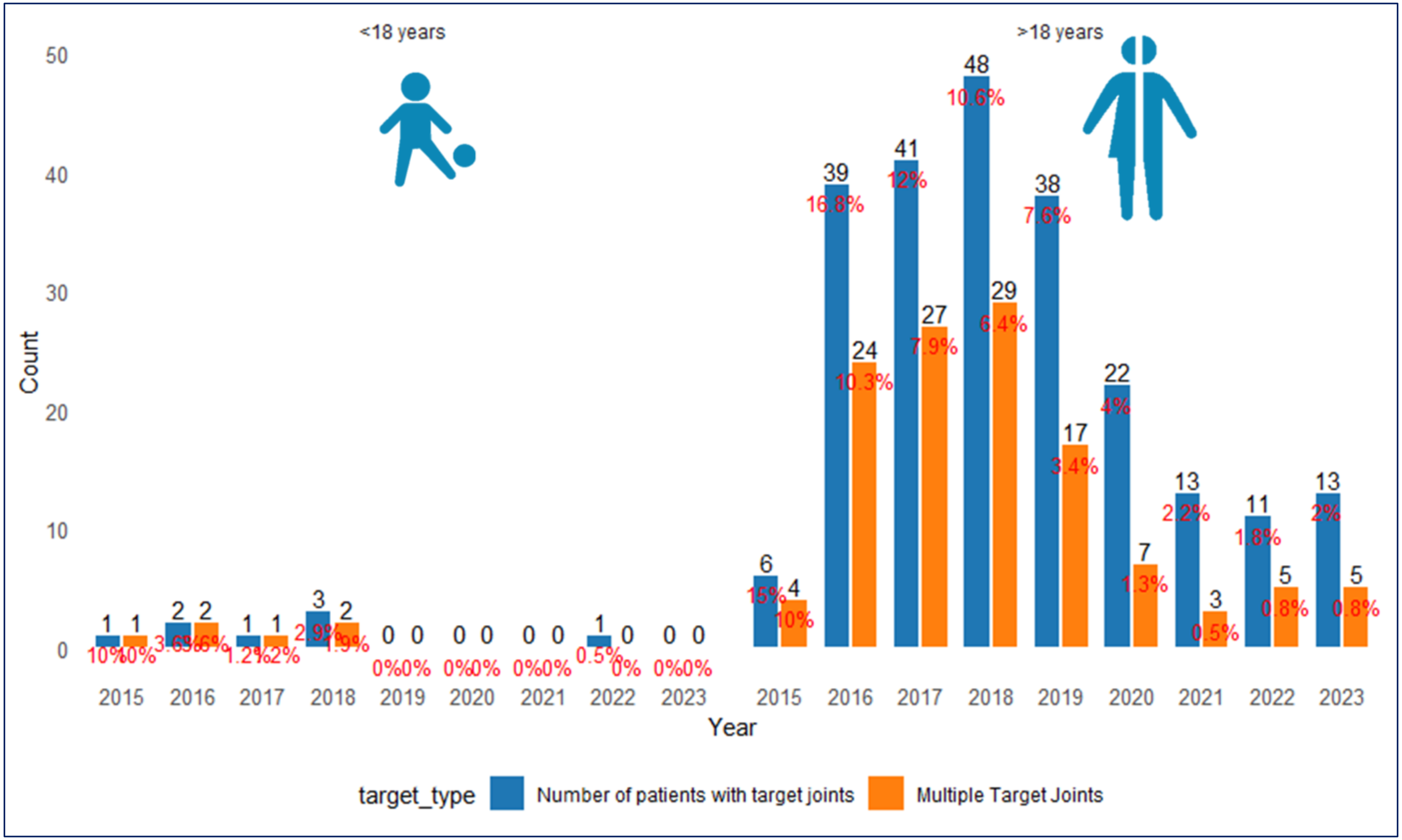

Supplementary Figure 3: Distribution of study centres for adults and children within Switzerland.

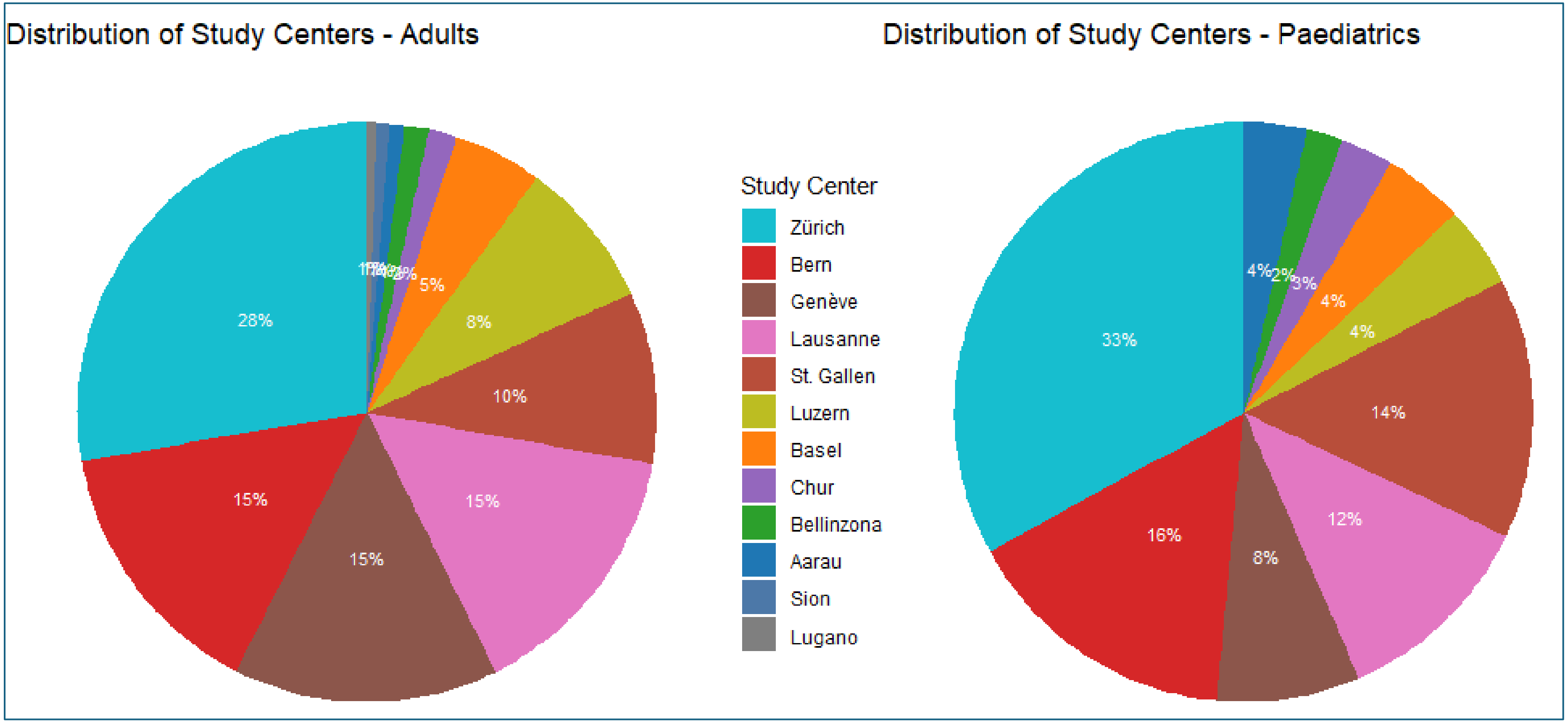

Supplementary Figure 4: Patients with hereditary bleeding disorders receiving anticoagulation or anti-platelet therapy. Indication (panel A) and product used (panel B).

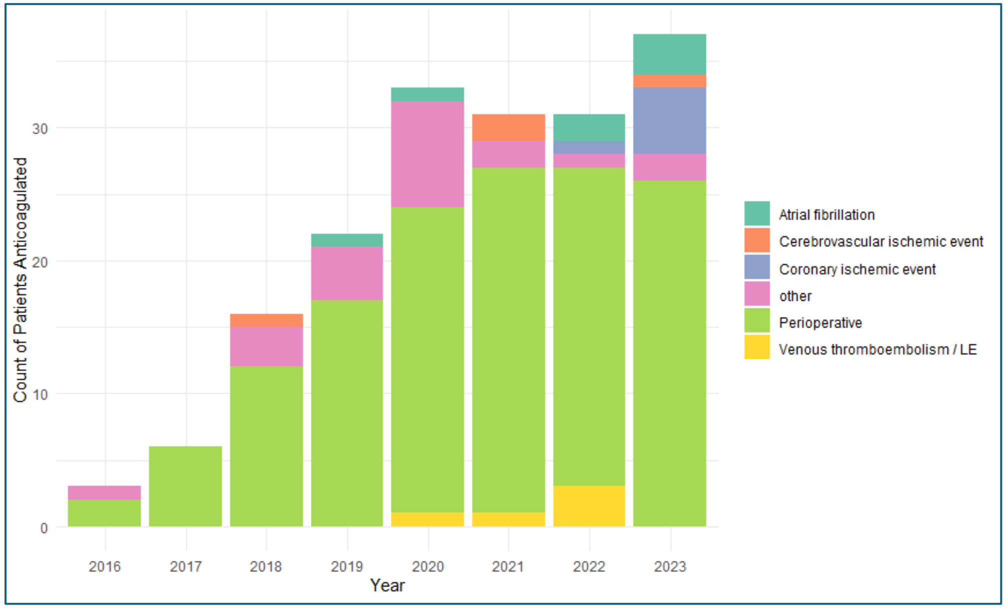

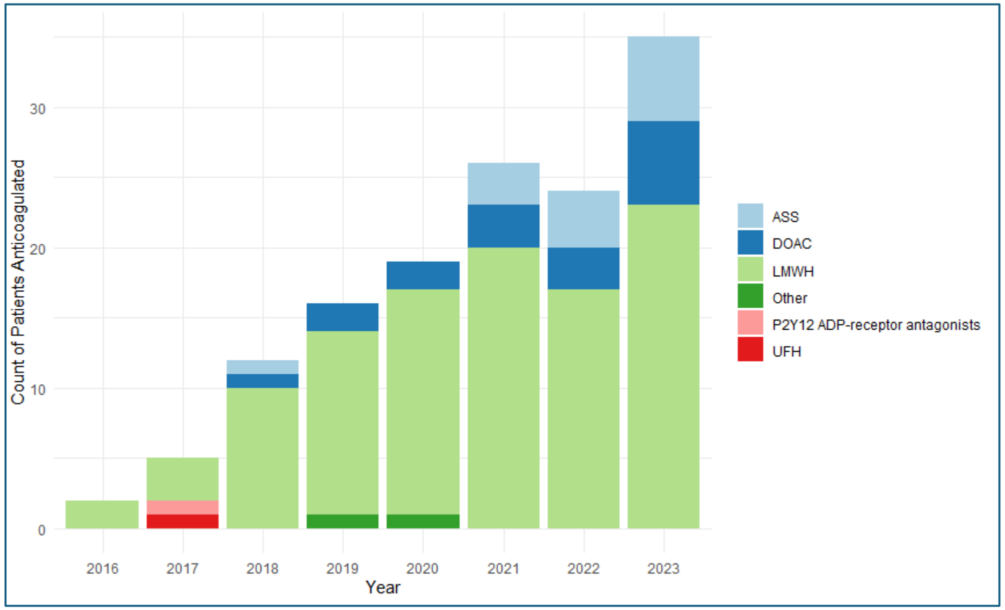

Supplementary Figure 5: Percent of patients with treated bleeds by year and by bleeding disorder

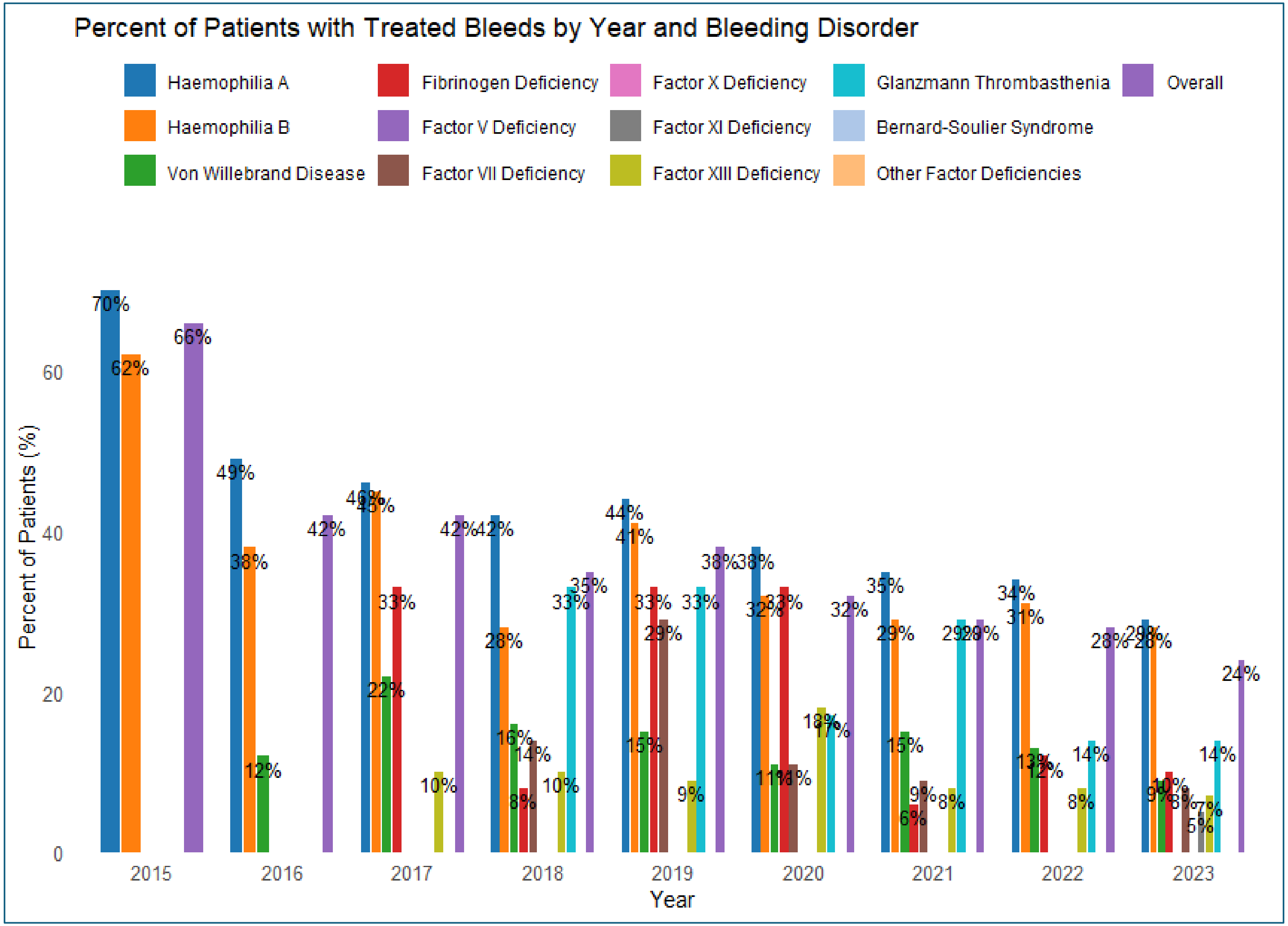

Supplement: Supplementary file 1 — Supporting Information Figure S1: Percentage of SHR patients on prophalyxis (left panel) or treated on demand (right panel) in 2023. Supporting Information Figure S2: Number of patients with haemophilia A and B with one or multiple target joints in children and adults. Supporting Information Figure S3: Distribution of study centres for adults and children within Switzerland. Supporting Information Figure S4: Patients with hereditary bleeding disorders receiving anticoagulation or anti‐platelet therapy. Indication (panel A) and product used (panel B). Supporting Information Figure S5: Percent of patients with treated bleeds by year and by bleeding disorder. [file HAE-32-923-s001.pdf]
